# Effect of Heating on Physicochemical Property of Aerosols during Vaping

**DOI:** 10.3390/ijerph19031892

**Published:** 2022-02-08

**Authors:** Tae-Jun Ko, Shin Ae Kim

**Affiliations:** Department of Innovation and Technology Research, ADA Science & Research Institute, American Dental Association, Gaithersburg, MD 20879, USA; kot@ada.org

**Keywords:** e-cigarette, aerosol properties, nicotine, heavy metal, toxicology

## Abstract

Many electronic cigarette manufacturers have offered different types of “high-end mods” that allow for controlled heating of the e-liquid. However, the controlled heating condition can drastically alter the inhaled aerosols’ physical properties and chemical substances, causing potential health risks. To investigate the contribution of heating on aerosol properties, we used four common power settings in the mods to conduct a physicochemical analysis. Our data showed that the aerosol mass and nicotine content in the aerosols increased at high power. Additionally, high power led to aerosolization of a viscous component in the e-liquid, increasing the viscosity of aerosol. However, the pH of the aerosol was constant regardless of the applied power. In addition, high-power operation made nicotine prone to oxidation, resulting in the color of the aerosol turning yellow. Lastly, we demonstrated that e-cigarette aerosol could contain various metals, including aluminum, arsenic, cadmium, chromium, copper, iron, magnesium, nickel, lead, and zinc. Even though these metal contents proportionally increased with the power setting, they remained far below the recommended exposure limits. Our finding demonstrates that the heating conditions of the e-cigarette change the physicochemical properties of the aerosols and their metal contents, thereby possibly affecting users’ oral and respiratory systems.

## 1. Introduction

E-cigarettes are electronic nicotine delivery systems (ENDS) designed to produce aerosols for vaping. To produce aerosols, a heating coil embedded in the e-cigarette heats up using Joule heating to vaporize e-liquids that consist of propylene glycol (PG), vegetable glycerin (VG), nicotine, and flavoring agents [1]. In contrast to traditional combustible tobacco, the e-cigarette only vaporizes aerosols, which are generally believed to be a less harmful alternative. For this reason, the e-cigarette is being chosen as a smoking cessation option. Thus, it has been gaining remarkable popularity among people who aim to quit or reduce tobacco smoking. According to recent statistics reported by the World Health Organization, the number of e-cigarette users has rapidly increased from 7 million in 2011 to 41 million in 2018 worldwide [2,3]. A similar trend is observed in the United States, with 3.6 million youth reporting using e-cigarettes in 2020 [4].

Despite the recent popularity, the potential long-term health risks of e-cigarettes are not fully understood, and studies are still ongoing. Many studies have reported that flavorants in e-liquids can produce various toxic chemicals, such as diacetyl, acetaldehyde, acrolein, and formaldehyde, which can potentially lead to cardiovascular and lung diseases [5,6,7]. In response to the youth epidemic of e-cigarettes and the potential toxicity of flavoring reagents, on 6 February 2020, the Food and Drug Administration (FDA) implemented a policy prioritizing enforcement against the manufacture, distribution, and sale of certain unauthorized flavored prefilled pod or cartridge-based e-cigarettes (excluding tobacco or menthol flavors).

Even though the emphasis on developing policy and regulations on e-liquid and flavoring reagents, limited efforts have focused on the heating conditions in vaping devices that can affect the inhaling aerosol properties. In fact, recently developed modifiable e-cigarette devices or “mods” provide multiple power setting options for the user to inhale different forms of aerosols. For example, a few typical mods on the market operate by heating sub-ohm coils that can generate various output power with an upper limit of 400 W and 315 °C. Thus, these mods are gaining more popularity because they offer various vaping experiences with customizable power and temperature settings [8]. However, studies are accumulating to show various causal effects from high power or high temperature during vaping: (1) creation of harmful volatile carbonyl compounds (e.g., acetaldehyde, acrolein, and formaldehyde) [7,9], (2) emitted aerosol particle sizes that may affect pulmonary deposition and absorption behavior [10,11,12], (3) formation of free radicals which can lead to chronic obstructive pulmonary disease or cardiovascular disease [13,14], and 4) generation of toxic heavy metals in aerosols [15,16].

To further investigate the influence of the heating conditions in vaping, we assessed the spatiotemporal temperature change of an e-cigarette device and conducted an aerosol correlation study using various heating conditions. We measured the physicochemical properties of the aerosol, such as mass, pH, viscosity, color, and metal contents. Lastly, we quantitatively analyzed the potential health risk based on calculating daily exposure limits of metals.

## 2. Materials and Methods

### 2.1. E-Liquid Preparation

E-liquids tested in all experiments were prepared following the literature [17]. A base solution of flavor-free reference e-liquid was prepared by mixing propylene glycol (>99.5%, CAS No. 57-55-6, Sigma Aldrich, St. Louis, MO, USA) and vegetable glycerin (>99.5%, CAS No. 56-81-5, Sigma Aldrich, St. Louis, MO, USA) with a weight ratio of 30 and 70, respectively. Nicotine (≥99%, CAS No. 54-11-5, Sigma Aldrich, St. Louis, MO, USA) was added to the base solution to achieve 1 wt% nicotine-containing e-liquid. The mixed e-liquid contained in a tube was agitated using a tube rotator with 10 rpm for at least 1 h to accomplish complete homogeneity.

### 2.2. E-Cigarette Device Operation and Aerosol Collection

Two power-regulated mods (Proton, Innokin, Shenzhen, China; X Class, SX Mini, Dongguan Yihi Electronic Co., Dongguan, China) were used with sub-ohm vape tanks (iSub-B, Innokin, Shenzhen, China). The sub-ohm tanks consisted of a coil head with a 0.35 ohm mesh-type Kanthal coil, wood pulp and cotton wicking, and a 3.0 mL capacity e-liquid reservoir. To evaluate the impact of e-cigarette operation power on physicochemical properties of the generated aerosols, we used four different power settings (20, 40, 60, and 80 W) available in most types of mods. All experiments used puff topography consisting of a puffing duration of 4 s and an interval of 18 s with 1 L/min flow rate, which was determined based on recent statistical studies [18,19]. The mass of the generated aerosol was calculated by measuring the mass difference of the e-liquid in the tank before and after 100 puffs (10 puffs/session × 10 vaping sessions). To measure the aerosol physicochemical properties, we captured aerosols using 0.22 μm glass fiber syringe filters (EZFlow^®^ HP Syringe Filter, Foxx Life Sciences, Salem, NH, USA) connected to the mouthpiece of the e-cigarette device, and the captured aerosol was collected in the container as depicted in Figure S1.

### 2.3. Temperature Measurement

Type-K thermocouples connected to a multichannel thermometer (TC0521, PerfectPrime, Barbican, UK) were used to simultaneously measure the temperature of multiple locations on the e-cigarette device (i.e., the center of the coil head, the inside and outside of the mouthpiece, the glass tube, and the base). The temperature of each part was recorded continuously for 10 puffs. In addition, an infrared (IR) thermal camera (Ti300+, Fluke Corporation, Everett, WA, USA) was utilized to provide a complementary visualization of the temperature distribution around the heating coil (top view) and the e-liquid containing sub-ohm tanks (side view) during device operation.

### 2.4. Gas Chromatography-Mass Spectrometry (GC-MS) Analysis

The nicotine contents in collected aerosol samples were measured by gas chromatography (GC, Clarus 680, PerkinElmer, Waltham, MA, USA) and mass spectrometry (MS, Clarus SQ 8C, PerkinElmer, Waltham, MA, USA) equipped with a capillary column (Elite-5MS, N9316282, PerkinElmer, Waltham, MA, USA). Detailed testing conditions were as follows: inlet temperature: 210 °C, carrier gas flow: 1.43 L/min, split: 1:5, temperature ramp: initial 40 °C, hold 3 min, 6 °C/min to 300 °C, hold for 3 min, total analysis time: 49.33 min. For quantitative nicotine analysis, 5 μL of 150 ppm Nicotine-d3 (≥99% deuterated forms, CAS No. 69980-24-1, Sigma Aldrich, St. Louis, MO, USA) was added as an internal standard (IS) solution with a non-coring needle to each 22 mL vial containing the collected aerosols, and then the nicotine contents were quantitated by comparison to the IS response. Prior to the measurement, we obtained nicotine peaks from four e-liquid samples to validate the reproducibility and confirmed that the relative standard deviation (RSD) of nicotine peak intensities was 2.8, verifying the reliability.

### 2.5. Viscosity Measurement

The viscosity of the e-liquid and collected aerosols was measured using a microfluidic viscometer (microVISC-m, Rheosense Inc., San Ramon, CA, USA). At least 200 μL of each collected aerosol was injected into a microfluidic viscometer using company-provided pipettes for all viscosity measurements. The temperature of e-liquid and aerosols was managed by an external temperature controller (Rheosense Inc., San Ramon, CA, USA) and maintained at 37 °C, the same as human body temperature.

### 2.6. pH Measurement

The pH of e-liquid and collected aerosols was measured using a microelectrode (accumet™ Micro Glass Mercury-Free Combination Electrodes, Fisher Scientific, Waltham, MA, USA). Before the measurement, the pH probes were calibrated with buffer solutions (pH 4.0, 7.0, and 10.0) according to the manufacturer’s instructions. The collected aerosols contained in glass vials were heated to 37 °C using a hotplate, and then pH values were recorded for 1 min until presenting stabilized pH values.

### 2.7. Color Assessment

Color readouts were performed on the collected aerosol solutions using a CIELAB-based colorimeter (WR10QC, Shenzhen Wave Optoelectronics Technology, Shenzhen, China) with a 4 mm aperture diameter. At least 300 µL of aerosol was captured by glass fiber filters and collected in a glass vial having a bigger diameter than the aperture of the colorimeter. Three color parameters based on CIELAB color space established by the “Commission Internationale de l’Eclairage (CIE)” were measured under D65 illumination; L* (lightness, 0 = black, 100 = white), a* (red to green, +a = red, −a = green), and b* (yellow to blue, +b = yellow, −b = blue). In addition, these measured L*, a*, and b* values were converted to XYZ coordinates based on CIE XYZ to calculate the yellowness indices (*YI E*313), which are expressed by the following Equation (1) [20,21]:

(1) $$YI\;E313=\frac{100}{Y}\left(1.3013X-1.1498Z\right)$$

### 2.8. Metal Concentration in Collected Aerosol Using Inductively Coupled Plasma-Mass Spectrometry (ICP-MS)

Aerosols captured by glass fiber filters were used for metal concentration analysis. For sample preparation, 200 µL of collected aerosols were diluted with 10 mL of 2% nitric acid solutions prepared by mixing distilled water and 70% nitric acid (CAS No. 7697-37-2, Sigma Aldrich, St. Louis, MO, USA). The metal contents for 14 metals, including silver (Ag), aluminum (Al), arsenic (As), cadmium (Cd), cobalt (Co), chromium (Cr), copper (Cu), iron (Fe), magnesium (Mg), manganese (Mn), nickel (Ni), lead (Pb), antimony (Sb), and zinc (Zn) were analyzed using inductively coupled plasma-mass spectrometry (ICP-MS; PerkinElmer NexION 300D ICP Mass Spectrometer with S10 autosampler, Waltham, MA, USA). The method used completed four replicates (1 reading per replicate; 40 sweeps per reading) with dual detector processing for analytes ^109^Ag, ^27^Al, ^75^As, ^111^Cd, ^59^Co, ^52^Cr, ^63^Cu, ^57^Fe, ^24^Mg, ^55^Mn, ^60^Ni, ^208^Pb, ^123^Sb, and ^67^Zn. Three samples per case were analyzed for analyte content (µg/L) to obtain the averages and standard deviation values. The limit of detection was 2.5 ppb for each metal.

The weight of generated metal per puff was calculated according to Equation (2) below.

(2) $$m_{me/puff}=\frac{m_{me/200\mu L}}{N_{200\mu L}}=\frac{C_{me}\times m_{sol/200\mu L}}{N_{200\mu L}}$$

In Equation (2), $m_{me/puff}$ is the weight of metal generated in one puff, $m_{me/200\mu L}$ is the weight of metal contained in 200 µL of aerosol, $N_{200\mu L}$ is the required number of puffs to generate 200 µL of aerosol, $C_{me}$ is the metal concentration, and $m_{sol/200\mu L}$ is the weight of 200 µL of aerosol solution. The daily metal inhalation by e-cigarette vaping (DMIE) was calculated considering the median number of daily e-cigarette puffs (i.e., 140 puffs) [18], and we further compared the DMIE to the recommended exposure limits (RELs) advised by the National Institute of Occupational Safety and Health (NIOSH) to assess potential health risks linked to excessive metal inhalation.

The RELs:NIOSH values are provided in a unit of the weight per space volume (i.e., mg/m^3^) and were converted to daily base recommended values (daily exposure limit, DEL) by multiplying the inhalation rate and the time-weighted average (8 h) to the RELs:NIOSH, as expressed in the following Equation (3).

DEL (mg) = (REL:NIOSH, mg/m^3^) × (0.83 m^3^/h) × (8 h) (3)

To note, we adopted the moderate activity inhalation rate (20 m^3^/day or 0.83 m^3^/h) and the time-weighted average (8 h/day), which were suggested by the European Medicines Agency and the NIOSH, respectively [22].

### 2.9. Chemical Composition Analysis on the Heating Coil and the Coil Head

Chemical compositions of the heating coil and the coil head were analyzed using a scanning electron microscope coupled with energy dispersive X-ray spectroscopy (SEM–EDS; JSM-IT500, JEOL Tokyo, Japan). Samples were mounted on an aluminum disk using carbon tape. An EDS spectrum was obtained simultaneously with the SEM images. The SEM-EDS was used with an accelerating voltage of 15 keV, pressure of ~7 × 10^−6^ Torr (high-vacuum mode), and 2000 magnifications with a working distance of 8–11 mm. The EDS spectrum and weight percent of each metal present in the heating coil and the coil head were obtained.

## 3. Results

### 3.1. Temperature Output via Various Power Settings

**Figure 1:**
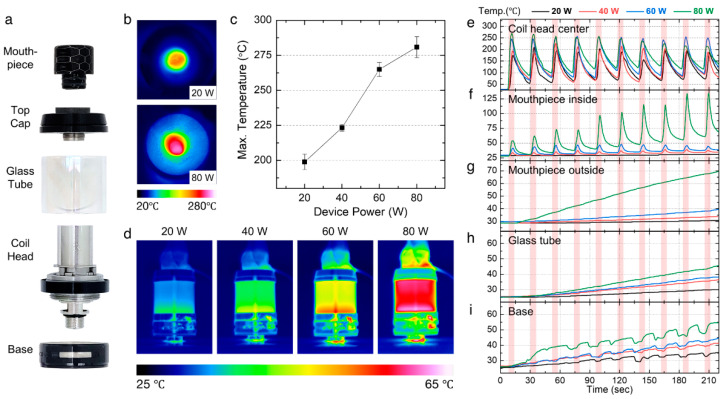
Spatial and temporal temperature distribution of the vape tank. (**a**) Components of the vape tank. (**b**) Top-view IR images showing the temperature of heated e-liquids at 20 and 80 W power settings. (**c**) Maximum temperatures of e-liquid measured using an IR camera during device operation with respect to the operating power. (**d**) Side-view IR images showing heat distribution throughout the tank at a power range from 20 to 80 W. (**e**–**i**) Temperature profiles measured using thermocouples during 10 puffs at a power range from 20 to 80 W at (**e**) the center of the coil head, (**f**) inside and (**g**) outside of the mouthpiece, (**h**) the glass tube outside, and (**i**) the base outside. One puff consisted of a puff duration of 4 s (highlighted in red in Figure 1e–i) and an interval of 18 s.

High-end mods allow the operation of power and temperature for the users to choose different vaping conditions based on their taste preferences [6]. To understand the correlation between the power setting and the temperature distribution of an e-cigarette device, we firstly assessed the spatial and temporal temperature distribution of a vape tank that consisted of the mouthpiece, the top cap, the glass tube, the coil head, and the base (Figure 1a) using an IR camera with different power settings, i.e., 20, 40, 60, and 80 W. Figure 1b shows the top view of the tank temperature distribution without the mouthpiece during device operation at 20 and 80 W. At 80 W, the high temperature of the heated coil increased the temperature of the entire tank. As shown in Figure 1c, the maximum e-liquid temperature measured by the IR camera increased from 198.9 to 280.8 °C as the power increased from 20 to 80 W. As shown in Figure 1d, the side view IR images that indicate overall tank temperatures also increased as the operation power increased.

We further measured the temperature profiles at five locations of the e-cigarette device (center of the coil head, inside and outside of the mouthpiece, glass tube surface, and the base surface) using thermocouples during operation. To measure the temperature of generated aerosol indirectly, we placed a thermocouple in the middle of the heating core inside the coil head and measured the temperature during 10 puffs. As shown in Figure 1e, the generated aerosol temperature peaked as the device turned on and gradually decreased upon cooling. The maximum peak temperatures shown in the temperature profiles were 218.9, 232.6, 260.8, and 271.9 °C as the power increased from 20 to 80 W, presenting a similar trend with the temperature values measured using the IR camera shown in Figure 1c.

To assess the temperature increase of the aerosol at the contact site to the user, we further measured the temperature profile of the inside and the outside of the mouthpiece. Figure 1f shows the temperature of the inside of the mouthpiece during 10 puffs. The peak temperatures for each puff were well-maintained during the different power settings. The maximum peak temperature at 20 W was 30.8 °C, and it showed a low level of deviation (± 1 °C) during 10 puffs. The maximum peak temperature at 60 W was 48.9 °C, and it was also kept at a low deviation, suggesting the mouthpiece temperature inside was well-managed without any substantial temperature increase. However, for 80 W, the peak temperature continuously increased during the 10 puffs and reached 144.3 °C after the tenth puff. Our data showed that the heat from aerosols was heating the mouthpiece. As for the outside of the mouthpiece where the user’s mouth directly contacts, the temperature gradually increased as the power increased (Figure 1g). The increasing tendency was steady, but the maximum temperature was much lower than that of the inner mouthpiece, e.g., 48.9 °C inside and 39.3 °C outside (similar to human body temperature) under 60 W. However, the temperature of the outside of the mouthpiece significantly increased under 80 W up to 69.6 °C after 10 puffs, indicating that excessive heat with high temperature at the inside of the mouthpiece also led to temperature rise at its outside surface.

The temperatures of the glass tube and the base were also measured (Figure 1h,i). In both cases, the measured temperatures gradually increased during 10 puff cycles at all power settings. Similar to the results from other parts, higher power settings resulted in higher temperatures. The glass tube and the steel base were heated to 45.7 and 54.7 °C at 80 W, which were lower than the maximum temperature of the mouthpiece (i.e., 144.3 °C for inside and 69.6 °C for outside at 80 W) because the glass tube and the base were not exposed to the aerosol stream.

### 3.2. The Mass of Aerosol and Nicotine Correlates with Heating

**Figure 2:**
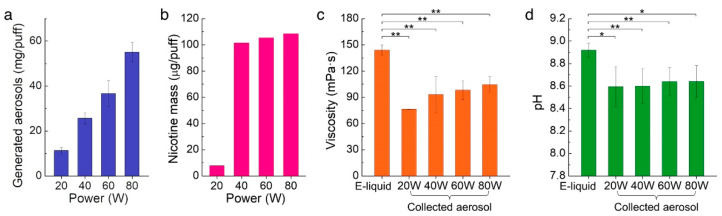
Measurement of physical and chemical properties of collected aerosols at the power range from 20 to 80 W. (**a**) Generated aerosol mass per puff, (**b**) nicotine mass per puff, (**c**) viscosity of e-liquid and collected aerosols, and (**d**) pH values of e-liquid and collected aerosols. In (**c**,**d**), *: *p*-value <0.05, **: *p*-value <0.01.

In order to assess the effect of vaping power on aerosol production, we measured the weight difference of the e-liquid before and after vaping to calculate the generated aerosol mass at different operation power. We averaged the mass of the produced aerosols per puff and plotted the mass based on the operation power. As shown in Figure 2a, the averaged mass of the aerosol proportionally increased with the power. The data indicated that more e-liquid vaporized to aerosol at a higher operation power.

Next, we further analyzed the nicotine concentrations in the aerosol at different powers using GC-MS analysis. As shown in Figure 2b, the nicotine mass contained in the aerosols at 20 W was only 7.86 µg/puff, which was significantly lower than that at the other power levels. This was because the temperature at 20 W (~198.9 °C) was not enough to vaporize the nicotine (boiling point, T_b_ = 250 °C) from the e-liquid. At 40 W or higher, the energy needed to aerosolize the nicotine compounds was reached so that the nicotine present in aerosol proportionally increased as power increased. The results suggest that both the total mass of the aerosol and the nicotine mass increased proportionally with increasing power, but the nicotine only vaporized sufficiently above 40 W [23].

### 3.3. High Heat Induces High Aerosol Viscosity

To investigate the effect of heating on the inhaled aerosol viscosity, we measured the viscosities of the collected aerosols at 37 °C, which is the temperature of the human body. The viscosity of our reference e-liquid was measured to 144 mPa·s, as shown in Figure 2c. The aerosol viscosities were lower than that of the e-liquid, but in a range between the viscosities of PG (22.8 mPa·s at 37 °C) and VG (301.6 mPa·s at 37 °C). In general, higher operation power yielded higher viscosity values due to the increased VG ratio in the aerosol. At low power (20 W), the temperature of the aerosol was slightly higher than the boiling point of PG (T_b_ = 188.2 °C) but did not reach the boiling point of VG (T_b_ = 290 °C). Therefore, PG, which has a relatively low viscosity, was predominantly vaporized at 20 W. However, the viscosity of the aerosol gradually increased as the amount of vaporization of VG increased at higher operation powers.

### 3.4. Aerosol pH Is Constant Independent of Heating Levels

To investigate the pH change during heating, we collected aerosols and measured pH levels with a microelectrode. As shown in Figure 2d, the pH value of the e-liquid was constantly higher than the pH of the aerosol by pH 0.2–0.4. However, the pH of the aerosol did not change in various operation power settings. The average pH values of the captured aerosols were uniformly measured in the range of pH 8.4–8.8, which was within the range of previously reported pH values [24]. It is known that nicotine mainly exists in its unprotonated form that is favorable for nicotine absorption through biological membranes as well as lung deposition under basic conditions (i.e., pH ≥ 8) [12]. Therefore, the nicotine in the aerosol still predominates in the unprotonated form, and it is expected that the change in nicotine absorption is negligible as the operation power increases.

### 3.5. High Heat Skews Aerosol to Yellow Color

**Figure 3:**
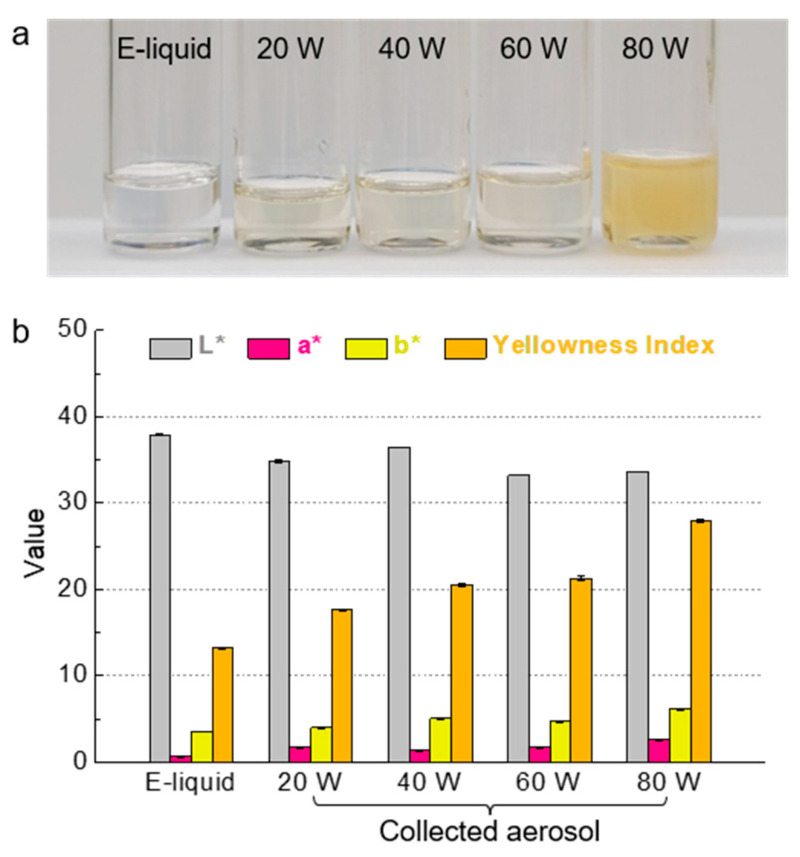
(**a**) Photo of the e-liquid and collected aerosols at a power range from 20 to 80 W. (**b**) Color assessment results presenting CIELAB values (L*, a*, and b*) and calculated yellowness indices.

In order to investigate the potential effect of vaping power on tooth discoloration, we measured the color of collected aerosols generated under different operation power settings. Figure 3a shows the color of the e-liquid and collected aerosols generated at 20–80 W. In visual observation, the color of the reference e-liquid containing 1% nicotine was clear. However, the color of the aerosol began to change from clear to yellow as the operation power increased. The collected aerosol solution generated at 20 W looked visually similar to the e-liquid, while the aerosol solution generated at 80 W was noticeably more yellow.

For more detailed numerical evaluation, we evaluated the color of the collected aerosols using a colorimeter under D65 illumination, which measures L*, a*, and b* values based on the CIELAB coordinate. Our data show that as the operation power increased, the L* value generally decreased and the a* and b* values increased (Figure 3b and Table 1). In particular, enhanced yellowness is known to be associated with a drastic increase in the b* value. To quantify the yellowness, the yellowness index (YI) was calculated by Equation (1). As presented in Table 1, the YI, which was 13.2 with the e-liquid, increased to 17.6 with the aerosol at 20 W, and further increased to 27.9 at 80 W.

### 3.6. Heating Can Increase Harmful Metals in Aerosol

**Figure 4:**
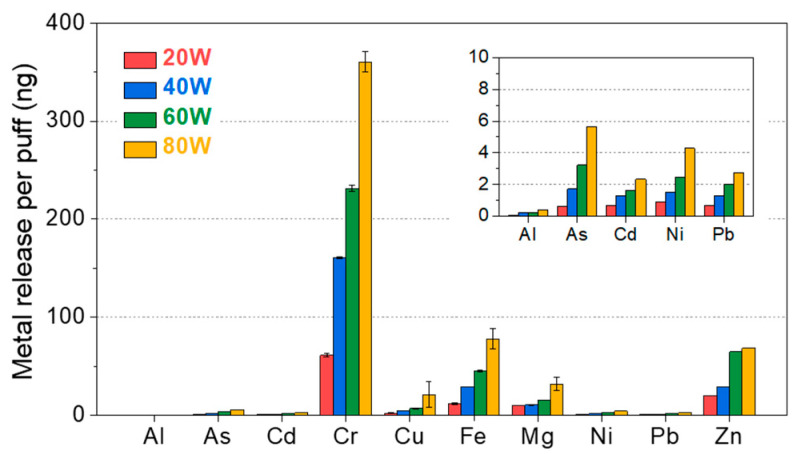
Metal release amount per puff derived from metal concentration in collected aerosols.

To quantify the effect of heating on the metal concentrations in aerosols, we measured 14 selected metal concentrations (Ag, Al, As, Cd, Co, Cr, Cu, Fe, Mg, Mn, Ni, Pb, Sb, and Zn) from the collected aerosols (200 µL) using ICP-MS at different power settings. Then, the metal concentrations were converted to the weight of generated metal per puff according to Equation (2) to reflect the metal intake per puff. The result showed that Al, As, Cd, Cr, Cu, Fe, Mg, Ni, Pb, and Zn were detected in all aerosol samples regardless of power (Figure 4). The release of those metals gradually increased as the operation power increased. This tendency suggests that higher heating could cause more metal particle release. Other metals such as Ag, Co, Mn, and Sb were not found in all samples. It is noted that other parts used in the aerosol collection (e.g., glass fiber filter, rubber tube, and polypropylene tube) were confirmed not to contain or contain a negligible level of the 14 selected metals, allowing us to exclude the possibility of metal influx from these parts (see Figure S2 in the Supplementary Material).

To assess the potential health risk caused by metal inhalation, we converted the obtained metal release per puff to the metal release per day (i.e., DMIE) by multiplying the number of median daily e-cigarette puffs, i.e., 140 puffs [18]. We then compared the DMIEs to the DELs using Equation (3). As shown in Table 2, the DMIE consistently increased as the power increased. Considering the median daily e-cigarette puff of 140 puffs, our calculation suggested that the DMIE of Al, As, Cd, Cr, Cu, Fe, Mg, Ni, Pb, and Zn by e-cigarette vaping at 80 W could increase to 0.06, 0.79, 0.33, 50.49, 2.97, 10.88, 4.49, 0.60, 0.39, and 9.59 μg. Even though with this drastic increment, the DMIEs at 80 W were still lower than the calculated DELs for 14 kinds of metals, as shown in Table 2. For example, the DMIE of Cr, which leached into aerosol the most among the 14 kinds of metals with 80 W, corresponded to only 1.5% of the calculated DEL. This result suggests that the amounts of metals possibly inhaled from e-cigarette vaping may not directly cause health concerns.

**Figure 5:**
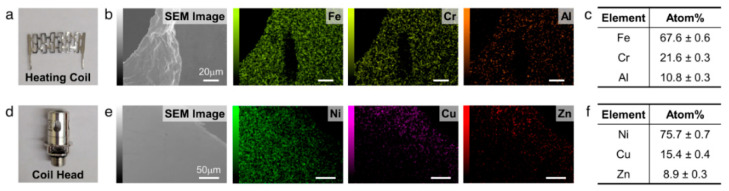
(**a**) A photo of a mesh-type heating coil. (**b**) SEM image and EDS elemental maps (Fe, Cr, and Al) of the heating coil. (**c**) The atomic concentrations of the heating coil obtained from the EDS analysis (**b**). (**d**) A photo of a coil head. (**e**) SEM image and EDS elemental maps (Ni, Cu, and Zn) of the surface of the coil head. (**f**) The atomic concentrations of the coil head obtained from the EDS analysis (**e**).

In order to trace the origin of the metal elements, we measured the chemical compositions of the heating coil and the coil head using SEM-EDS, as shown in Figure 5. According to the EDS analysis (Figure 5c,f), the heating coil mainly consisted of Fe, Cr, and Al, while the coil head was found to mainly comprise Ni, Cu, and Zn. It was noted that a large amount of Fe and Cr, which are components of the heating coil, were detected from the aerosols generated from all power settings. However, the metal elements comprised by the coil head (i.e., Ni, Cu, and Zn) were primarily found in the aerosol generated at high power. These results suggest that high temperature can affect the leaching of metals not only from the heating coil but also from the coil head. However, the sources of As, Cd, Pb, and Mg, which were not detected in the EDS analysis of the heating coil and the coil head, were still unknown, but it was assumed that a tiny amount of those elements were included in the heating coil or the coil head.

## 4. Discussion

To study the effect of heating on aerosols, we characterized the physicochemical properties of aerosols generated from flavor-free e-liquid reference materials at four different power settings. Our temperature results showed that the maximum temperatures of each component, except the coil head, continued to increase during the repeated puffs due to the excessive heat that could not be fully cooled between puffs (Figure 1). The high temperature of the mouthpiece (i.e., 144.3 °C for inside and 69.6 °C for outside at 80 W after 10 puffs) can cause burns on the lip and oral epithelium since the mouthpiece directly contacts the e-cigarette user. It is known that the basal layer of the epidermis can be burned at a temperature of 44 °C or higher, and the damage rate is accelerated at over 70 °C [25]. Based on our data, an acceptable power range to prevent burning on the lip and the oral epithelium is below 60 W.

Moreover, the viscosity of the aerosols can be an additional factor contributing to health risk. PG and VG in the e-liquid solution have a relatively high viscosity. In particular, VG has a very high viscosity (e.g., 301.6 mPa·s at 37 °C), thereby, the generated aerosols with high VG ratios at high power settings are likely to adhere to exposed surfaces, including soft tissues in the oral cavity and airway, as well as hard tissues such as tooth surface [26]. These findings support our previous study that a higher amount of viscous aerosol attachment on tooth surface increased cariogenic potential by increasing the adhesion force of pathogenic bacteria such as *Streptococcus mutans* [27].

Recent studies have shown that e-cigarette vaping, as well as combustible tobacco smoking, can cause tooth discoloration by surface staining on enamel or dental restoratives [28]. In this regard, the color of aerosols that are products of e-cigarette vaping can strongly influence the discoloration behavior in a user’s teeth. In particular, the aerosol generated at high power renders a more yellowish color, and it has a higher viscosity that allows it to adhere to the tooth surface for a longer time, inducing the potential to accelerate tooth discoloration further.

The color change of the e-liquid is presumed and known to be affected by nicotine oxidation. Several studies reported that various byproducts of nicotine oxidation (e.g., cotinine, myosmine, anabasine, and nicotyrine) are produced during vaping, and in particular, nicotyrine was suggested to be closely connected to the color change of e-liquid [29,30]. However, the exact mechanism and criteria that cause the color change were not fully unveiled yet. Thus, future studies regarding how chemical reactions result in composition changes and how discolored aerosols contribute to tooth discoloration are expected to follow.

The presence of metal elements is an emerging issue in e-cigarette research due to the unknown health effects on e-cigarette users [22,31,32,33]. A recent clinical study demonstrated that e-cigarette users have certain levels of Ni and Cr in urine and saliva, suggesting the possibility of a causal relationship between e-cigarette usage and internal metal doses [34]. Some of the detected heavy metals were carcinogenic agents defined by the International Agency for Research on Cancer (IARC) with the following four classifications; group 1: carcinogenic to humans, group 2A: probably carcinogenic to humans, group 2B: possibly carcinogenic to humans, and group 3: not classifiable as to its carcinogenicity to humans. With a 5.9-fold increase at 80 W compared to that at 20 W, Cr is a group 1 carcinogen with an increased risk of lung cancer and respiratory disease [34]. In addition, other carcinogens, Ni (group 1 as a compound, and group 2B as a metallic form) and Pb (group 2B as a metallic form, and group 2A and 3 as a compound), were also detected 4.8- and 4.2-fold more at 80 W compared to 20 W, respectively. These carcinogen elements are known to cause serious respiratory disease, including lung and nasal sinus cancers [22]. Although in trace amounts, other well-known group 1 carcinogen heavy metal elements As and Cd were also detected more at high power.

The quality and quantity of chemical byproducts and metals in e-cigarette aerosol are closely related to the material composition of the vape tank (including heating coils and coil heads) and the conditions used to generate the aerosols [15,17]. In particular, metal elements in heating coils and coil heads leached into the e-liquid contained in the tank, as well as the generated aerosol. The leaching behavior has been known to be affected by operating conditions, e.g., temperature, pH, and reactivity or solubility between metal and liquid [35]. Since the leaching capacity and rate significantly increase as temperature rises [36], aerosols generated at higher temperatures induced by higher power are known to contain more metal elements.

This study has the following limitations. First, the operation power range was restricted. Recently, a few mods that operate at a more comprehensive power range up to 400 W (heating up to 315 °C) have been released on the market. Our study demonstrated the effect of heating temperature on aerosol properties within the temperature range from 198.9 to 280.8 °C. However, we could not explore the impact above 300 °C, which is expected to significantly affect several factors, such as viscosity and nicotine/metal concentration. Second, even though we revealed that metal leaching behavior is strongly related to temperature, our study was only conducted with Kanthal. To elucidate the causes and pathways of metal leaching in the e-cigarette vaping environment, further investigation using a variety of heating coils and coil heads containing different types of materials, e.g., Nichrome, stainless steel, or ceramic heater, will be required. At last, a precise chemical analysis to analyze the presence and levels of nicotine and related alkaloids should be followed.

## 5. Conclusions

In this study, we demonstrated that e-cigarette operation power settings heavily influence the temperature variation on vape tank configurations, amount of aerosol generation and nicotine contents, viscosity and color of aerosols, and metal concentration in aerosols. As the operation power increased, the heating temperature and the amount of generated aerosol increased, contributing to increasing overall aerosol inhalation per puff. The physicochemical properties of aerosols varied depending on the power settings. In particular, viscosity values were more affected depending on the operation power since VG, with high viscosity and a high boiling point, was mainly vaporized at high power. More importantly, metal release in aerosols was significantly increased at high operation power. Our comprehensive metal analysis suggests that the metals in the heating coil and the coil head could be emitted at higher levels at higher temperatures, increasing the carcinogenic potential. Based on our data, the variation in physicochemical properties caused by the vaping power may result in various deposition profiles in human organs, such as teeth, oral soft and hard tissues, respiratory tract, and lung tissues, which can also cause health risks.

In summary, our study informs the potential risks in using high temperature with vaping for oral and respiratory health. We believe our study provides a foundation for discussion on e-cigarette related regulation, vaping instruction for users, and designing future e-cigarette devices.

## Figures and Tables

**Table 1:** Color assessment of aerosols generated by e-cigarette with different power.

Sample	CIELAB			CIE XYZ			Yellowness Index (E313)
	L*	a*	b*	X	Y	Z	
E-liquid	37.94	0.64	3.53	9.64	10.06	9.75	13.23
20 W	34.85	1.71	3.96	8.19	8.42	7.98	17.61
40 W	36.44	1.29	5.05	8.93	9.24	8.47	20.46
60 W	33.20	1.71	4.72	7.43	7.63	7.00	21.26
80 W	33.66	2.55	6.11	7.73	7.85	6.84	27.90

**Table 2:** Daily metal inhalation by e-cigarette vaping (DMIE) and calculated daily exposure limits (DELs) for 14 metals.

Element	NIOSH:REL (µg/m^3^)	Calculated DEL (µg) ^1^	Daily Metal Inhalations by E-Cigarette (DMIE) (µg) ^2^				DMIE/DEL (%)			
			20 W	40 W	60 W	80 W	20 W	40 W	60 W	80 W
Ag	10	67	<LOD ^3^	<LOD	<LOD	<LOD	0	0	0	0
Al	5000	33,500	0.007	0.033	0.036	0.059	<0.001	<0.001	<0.001	<0.001
As	10	67	0.084	0.239	0.451	0.791	0.125	0.357	0.673	1.18
Cd	5	33.5	0.097	0.183	0.23	0.326	0.289	0.547	0.687	0.973
Co	50	335	<LOD	<LOD	<LOD	<LOD	0	0	0	0
Cr	500	3350	8.577	22.5	32.4	50.49	0.256	0.672	0.967	1.507
Cu	1000	6700	0.307	0.61	0.964	2.969	0.005	0.009	0.014	0.044
Fe	5000	33,500	1.672	4.068	6.343	10.88	0.005	0.012	0.019	0.032
Mg	10,000	67,000	1.399	1.485	2.177	4.492	0.002	0.002	0.003	0.007
Mn	1000	6700	<LOD	<LOD	<LOD	<LOD	0	0	0	0
Ni	15	100.5	0.126	0.214	0.347	0.599	0.125	0.213	0.346	0.596
Pb	50	335	0.093	0.184	0.281	0.386	0.028	0.055	0.084	0.115
Sb	500	3350	<LOD	<LOD	<LOD	<LOD	0	0	0	0
Zn	5000	33,500	2.811	4.035	9.064	9.589	0.008	0.012	0.027	0.029

^1^ Calculated DEL = (NIOSH: REL) × (moderate activity inhalation rate, 0.83 m^3^/h) × (time-weighted average, 8 h). ^2^ DMIE = (metal release per puff) × (number of median daily e-cigarette puffs, 140 puffs). ^3^ <LOD: Lower than the limit of detection of ICP-MS (2.5 ppb).

## Supplementary Materials

The following supporting information can be downloaded at: https://www.mdpi.com/article/10.3390/ijerph19031892/s1, Figure S1: A schematic of the experiment setup with the e-cigarette aerosol collecting system. Figure S2: Structural and chemical analysis on a glass fiber filter and rubber tube.
