# Supplementary material for: Effect of Heating on Physicochemical Property of Aerosols during Vaping
Source: Int J Environ Res Public Health. 2022 Feb 8;19(3):1892. doi: 10.3390/ijerph19031892 (PMC8835267; doi:10.3390/ijerph19031892)
Supplement: Supplementary file 1 [file ijerph-19-01892-s001.zip › ijerph-1509229-supplementary.pdf]

International Journal of
Environmental Research and Public Health

MDPI

Supplementary Material— Effect of heating on physicochemical property of aerosols during vaping

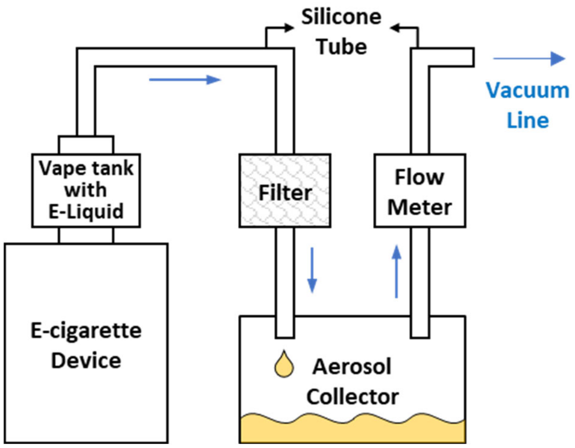

Figure S1. A schematic of the experiment setup with an e-cigarette aerosol collecting system.

a

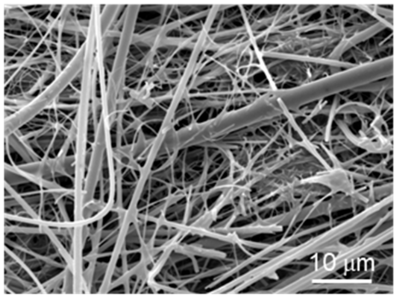

b

Element	Atom%
C	28.9
O	44.1
Si	18.2
Na	5.5
Al	2.0
K	1.3

c

Element	Atom%
Si	62.8
O	37.2

Figure S2. Structural and chemical analysis on a glass fiber filter and rubber tube. (a) An SEM image of a glass fiber filter surface. Atomic concentration analysis on (b) a glass fiber filter and (c) a silicone rubber tubing surface using EDS.
